# Intensity of perioperative analgesia but not pre‐treatment pain is predictive of survival in dogs undergoing amputation plus chemotherapy for extremity osteosarcoma

**DOI:** 10.1111/vco.12808

**Published:** 2022-03-21

**Authors:** Michael W. Nolan, Olivia C. Uzan, Noah A. Green, Susan E. Lana, B. Duncan X. Lascelles

**Affiliations:** ^1^ Department of Clinical Sciences, College of Veterinary Medicine North Carolina State University Raleigh North Carolina USA; ^2^ Comparative Medicine Institute North Carolina State University Raleigh North Carolina USA; ^3^ Comparative Pain Research and Education Center North Carolina State University Raleigh North Carolina USA; ^4^ Department of Clinical Sciences, College of Veterinary Medicine and Biomedical Sciences Colorado State University Fort Collins Colorado USA; ^5^ Flint Animal Cancer Center Colorado State University Fort Collins Colorado USA; ^6^ Translational Research in Pain Program, Department of Clinical Sciences, College of Veterinary Medicine North Carolina State University Raleigh North Carolina USA; ^7^ UNC School of Medicine Thurston Arthritis Center Chapel Hill North Carolina USA; ^8^ Center for Translational Pain Research, Department of Anesthesiology Duke University Durham North Carolina USA

**Keywords:** analgesia, cancer pain, cancer treatment pain, local anaesthesia, malignant osteolysis

## Abstract

The purpose of this bi‐institutional retrospective study was to determine whether, in dogs treated with limb amputation and adjunctive chemotherapy for osteosarcoma, oncologic outcomes are impacted by either: (1) baseline cancer pain severity, or (2) the approaches used for perioperative pain management. Data were extracted from the medical records of 284 dogs that underwent both limb amputation and chemotherapy (carboplatin and/or doxorubicin) between 1997 and 2017 for localized (non‐metastatic) osteosarcoma of the appendicular skeleton. Kaplan–Meier survival curves and Cox proportional hazard (PH) models were used to determine the impact that retrospectively scored baseline pain levels (high vs. low) and various analgesic and local anaesthetic treatments had on both metastasis‐free survival and all‐cause mortality. For the entire population, the median disease free interval and median overall survival times were 253 and 284 days, respectively. Baseline pain was rated as “low” in 84 dogs, and “high” in 190 dogs; pain severity had no detectable effect on either metastasis‐free survival or all‐cause mortality. When accounting for the potential influences of known prognostic factors, dogs treated with what was characterized as a high‐intensity perioperative analgesic plan (including both a non‐steroidal anti‐inflammatory drug [NSAID] and a bupivacaine‐eluting soaker catheter placed at the amputation site) had a higher probability of survival than dogs treated with a low‐intensity perioperative analgesic plan (neither an NSAID, nor a soaker catheter); the median overall survival times were 252 and 378 days, respectively (hazard ratio: 2.922; *p* = .020).

## 1 INTRODUCTION

A majority of dogs with extremity osteosarcoma display overt signs of pain.^1^ That pain is variably and typically incompletely responsive to standard orally administered pain medications; while treatments such as intravenously administered bisphosphonates, intrathecally administered resiniferatoxin, and radiotherapy offer more reliable and more durable analgesia, pain remains problematic due to its negative impact on quality of life.^1^, ^2^, ^3^, ^4^ Osteosarcoma‐associated pain may also influence prognosis. A recent retrospective study provides evidence that in dogs undergoing chemoradiotherapy for extremity osteosarcoma, high pain is predictive of short overall survival.^5^

It is unclear whether pain is simply a bellwether for early osteosarcoma‐associated death, or if there is a true mechanistic link between pain and cancer progression. One consideration is that the pro‐nociceptive ligands being produced during tumour progression and leading to generation of pain signals (e.g., nerve growth factor, endothelin‐1, prostaglandin E2, etc.) may also be promoting aggressive tumour behaviours.^6^, ^7^ Prognosis may also be influenced by both the pain caused by cancer treatment, and the manner in which that pain is managed. Experimentally, surgical pain can enhance retention of cancer cells in the lungs of rats.^8^ That effect can be mitigated with morphine.^9^ However, there is also concern that opioids themselves may hasten cancer recurrence or metastasis, either as a direct effect of the drugs, or perhaps opioid‐induced immunosuppression enables escape of cancer cells from immune surveillance.^10^, ^11^ Similar observations have been made in humans. It has been reported that after transurethral resection of superficial bladder cancer, recurrence rates are lower when spinal anaesthesia is used instead of general anaesthesia.^12^ Similarly, following open thoracotomy for resection of primary lung tumours, overall survival was longer in patients treated with a paravertebral block, versus intravenous patient‐controlled analgesia.^13^ These retrospective studies are limited by small sample size and failure to account for all potential confounders and known prognostic factors; there are also similarly designed retrospective studies, and even some well‐designed prospective investigations that fail to find an association between the analgesic/anaesthetic approach and oncologic outcomes.^14^, ^15^, ^16^ Thus, the potential for pain and pain management to influence oncologic outcomes may be restricted to certain malignancies, patient populations, and treatment approaches.

In dogs with non‐metastatic osteosarcoma, the most widely accepted definitive‐intent treatment is limb amputation followed by adjunctive chemotherapy (most often carboplatin and/or doxorubicin). In dogs treated as such, it remains uncertain whether baseline tumour‐associated pain severity is predictive of long‐term survival. It is also unknown whether surgical pain, or the choice of which perioperative analgesics are used to manage that pain, might influence outcomes. Therefore, the purpose of this study was to determine whether progression‐free or overall survival times are associated with either: (1) retrospectively assigned baseline pain severity scores, or (2) the types of analgesics and local anaesthetics that were used before, during, and in the first few days following surgical limb amputation.

## 2 METHODS

### 2.1 Data acquisition

A bi‐institutional retrospective study was performed. Institutional animal care and use committee approval was not required. For each included case, standard written pet owner consent for treatment was obtained; case management was at the discretion of the medical team managing the case at the time. Oncology accession logs (February 1997–September 2017) at two academic veterinary teaching hospitals were searched for cases in which limb amputation and at least one dose of adjuvant chemotherapy (carboplatin, doxorubicin, or a combination thereof) was used as treatment for histologically confirmed osteosarcoma in dogs that were free of both lymph node metastasis and pulmonary metastasis (as determined by three‐view thoracic radiographs). Dogs were excluded if they had pathologic evidence of lymph node metastasis; in cases for which cytology and/or histopathology was not performed, nodes were deemed “normal” whenever there was absence of palpable regional lymphadenopathy in recorded physical exam data. Cases were excluded if body weight at diagnosis was less than 15 kg.^17^ Cases were also excluded if there was lack of access to the histopathology report, inability to ascertain the nature of chemotherapy protocols, or insufficient follow‐up to ascertain overall survival time.

The following data were extracted from the medical records: breed, sex and neuter status, date of birth, body weight at diagnosis, tumour location, preoperative total serum alkaline phosphatase (ALP) concentration, preoperative absolute monocyte count, date of amputation, types of analgesics and local anaesthetic techniques used perioperatively, details of chemotherapy, date and site of metastasis, date of last follow‐up, and date and cause of death. A baseline pain score was retrospectively assigned as either “high” or “low”, as previously described.^5^ Classification of pain as “high” required documentation of a pain score of 3 or 4 out of 4, presence of a non‐weight bearing lameness, and/or description of moderate‐to‐severe pain in the physical examination; “low” pain was defined by a pain score of 0, 1, or 2 out of 4, weight‐bearing lameness, and/or description of absent to mild pain on physical examination.^18^ Pain scores, based on the Colorado State University Acute Pain Scale, were not assigned retrospectively; we only used contemporaneous observations that were made by the attending clinician and recorded in the medical record at the time of assessment. No attempt was made to record use of non‐pharmacologic analgesia strategies (e.g., acupuncture). Data were not collected regarding: histopathologic margin status, chemotherapy dosing or dose interval, or adverse effects of treatment.

### 2.2 Statistical analyses

Overall survival time (OST) was defined as the time from amputation to death. If lost to follow‐up, cases were censored at the time of last contact, and cases were also censored if alive at the time of analysis. Progression‐free survival (PFS) was defined as the time from amputation until identification of (radiographically or pathologically diagnosed) metastatic disease; subjects were censored if alive without evidence of tumour progression at the time of analysis, or if the cause of death was demonstrably unrelated to OS. Kaplan–Meier methods were used to calculate median PFS and OST with 95% confidence intervals (CI). Factors evaluated for potential predictive or prognostic value included: age; sex; body weight; tumour location (proximal humerus vs. other); treatment facility; preoperative ALP and monocyte count; baseline pain score (high vs. low); use of NSAIDs (yes/no) in the pre‐ or perioperative periods or after discharge; category of NSAID use (none vs. use during all periods [preoperative, perioperatively and post‐discharge]); use of non‐NSAID analgesics (none, single agent, multiple drugs [multimodal]) in the pre‐, intra‐, postoperative and post‐discharge periods; local anaesthetic usage (none, nerve or line block, epidural, or soaker catheter); and intensity of overall perioperative analgesic support, which was categorized as low (no NSAID at any time and either no local anaesthetic or only a simple line or nerve block) or high (an NSAID used during all periods [preoperative, perioperative and post‐discharge], plus a local anaesthesia‐eluting soaker catheter with or without other local anaesthetic usage). Preoperative NSAID use was determined from review of the medical history recorded at surgical admission. Perioperative NSAID use included any administration in the period beginning 24 h before surgery and ending at hospital discharge. For non‐NSAID analgesics, the preoperative period included any drug use recorded at surgical admission, or provided preoperatively while an inpatient. Intraoperative non‐NSAID analgesic use was determined from review of anaesthesia records, surgical reports and controlled drug logs; the postoperative period was from extubation until hospital discharge. For both NSAIDs and non‐NSAID analgesics, post‐discharge drug use included any medications recommended or prescribed at the time of postamputation hospital discharge. Compliance with post‐discharge medication administration was not assessed. Use of other adjunctive analgesics and sedatives (e.g., ketamine, dexmedetomidine, acepromazine) was also not assessed; this is because there was variable usage and essentially complete inability to understand from the medical records what decision‐making processes led to the prescription or discontinuation of these medications (and doses) in individual animals.

Univariate analyses were performed via the Log‐Rank test. Variables with *p* <0.20 were then entered into a multivariable Cox proportional hazard regression model. The ENTER method was used, with variables retained in the model if *p* <.05. For Log‐Rank testing, ALP and monocyte counts were dichotomized as < or ≥ the population median; monocyte counts were also assessed as being < or ≥ 400.^19^ For the multivariable model, ALP and monocyte count were assessed as continuous variables. Statistical significance was set at α = .05; analyses were performed using commercial software (SPSS version 26; IBM Corporation, Armonk, NY; and Prism version 8; GraphPad Software, San Diego, CA).

## 3 RESULTS

Due to limited availability of medical records, one institution in the western United States contributed case materials from February 1997 to December 2010 and the other (in the eastern United States) from January 2010 to September 2017. In total, 309 cases were identified; 284 were included and the remaining 25 cases were excluded because they were < 15 kg (*N* = 4), did not have available histopathology reports (*N* = 4), details of chemotherapy administration could not be confirmed (*N* = 16), or survival time could not be ascertained (N = 1). Of the included dogs, there were 145 castrated males, 130 spayed females, 5 sexually intact males, and 4 sexually intact females; the median age was 9 years (interquartile range: 4.5 years), and the median body weight was 38 kg (interquartile range: 15.6 kg). Dog breeds included mixed breed (62), Labrador Retriever (49), Rottweiler (39), Golden Retriever (32), Greyhound (23), German Shepherd (13), Great Pyrenees (11), Doberman Pinscher (7), Saint Bernard (6), Boxer (4), Malamute (4), Irish Setter (4), American Staffordshire Terrier (3), Mastiff (3), Akita (3), Newfoundland (3), Irish Setter (2), Husky (2), Cane Corso (2) and 1 each of the following: Dalmatian, Flat Coated‐Retriever, German Short‐haired Pointer, Golden Doodle, Irish Wolfhound, Leonberger, Plott Hound, Poodle, Rhodesian Ridgeback, Soft Coated Wheaten Terrier, Staffordshire Bull Terrier, and Weimaraner. For all dogs, the median PFS was 252 days (95% CI: 229–275) and the median OST was 284 days (95% CI: 208–298). Four cases were censored from the analysis of overall survival time; one was alive at 1217 days and 3 were lost to follow‐up at 248, 910 and 1307 days post‐amputation. For the PFS analysis, 92 cases were censored; 7 died of a cause that was demonstrably unrelated to osteosarcoma and 85 were alive without evidence of tumour progression at the time of analysis, or when lost to follow‐up.

Line and nerve blocks were performed with either lidocaine, bupivacaine, or a bupivacaine liposome injectable suspension (Nocita®; Elanco Animal Health, Greenfield, IN, USA). Epidurals were with lidocaine or bupivacaine, and with or without addition of morphine. Bupivacaine was the local anaesthetic instilled into the wound whenever a soaker catheter was used. For this analysis, we did not consider drug potency/efficacy, or dose; we simply recorded and considered the category of use of local anaesthetic. Bupivicaine liposomal injectable suspension was used for line and/or nerve blocks in a total of 10 dogs; 4 were in the group whose intensity of overall perioperative analgesic support was “low”, and none were in the group for which intensity was “high.” With regard to the soaker catheters, all dogs in which soaker catheters were used were prescribed 1–2 mg/kg bupivacaine (0.5%) infused once every 6–8 h. The drug was administered an average of 6.6 times, and the minimum number of doses received postoperatively was 3. A total of six dogs received between three and five doses of bupivacaine through the soaker catheter, five dogs received 6–8 doses, and five dogs received 9–12 doses.

**FIGURE 1:**
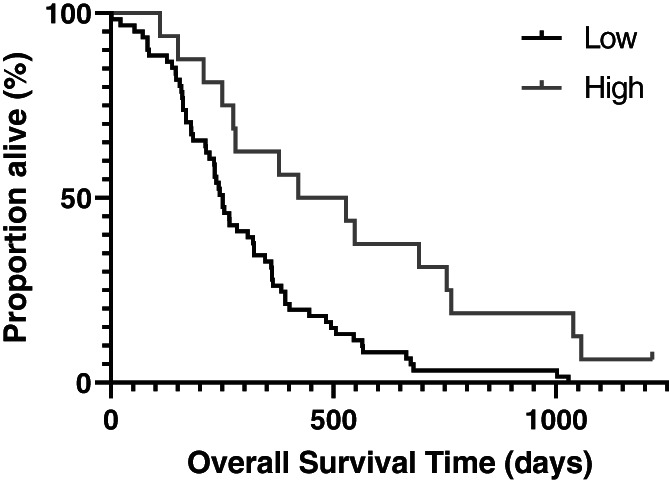
Kaplan–Meier graph of the overall survival time (OST) for dogs defined as having received a low (*n* = 61 dogs; median: 252 days; 95% confidence interval: 217–287 days) versus high (*n* = 16 dogs; median: 378 days; 95% confidence interval: 196–560 days) level of analgesic support in the time surrounding limb amputation (Log‐Rank *p* value = .008)

In univariate testing (results of which are summarized in Table 1), baseline serum ALP greater than or equal to the population median was significantly associated with shorter PFS; OST was shorter in dogs with proximal humeral tumours, serum ALP concentration at or above the population median, and low overall intensity of perioperative analgesic support (Figure 1). Factors included in the multivariable Cox proportional hazards regression model were: age, monocyte count, serum ALP concentration, tumour location, and perioperative NSAID use (i.e., variables with Log‐Rank *p* <0.20), as well as the experimental variables of interest: “baseline pain” and “intensity of overall perioperative analgesic support” (Table 2). In this assessment, baseline pain scores were not associated with survival. While the intensity of overall perioperative analgesic support was not associated with PFS, dogs receiving high intensity perioperative analgesic support did have higher odds of prolonged overall survival (hazard ratio: 2.922 [95% CI: 1.189 to 7.194]; *p* value: .020).

**TABLE 1:** Univariate analysis of the impact that various factors have on survival

Log‐rank testing of categorical variables		Progression‐free survival					Overall survival				
		*N*	Median	95% CI			N	Median	95% CI		
				Lower	Upper	Sig.			Lower	Upper	Sig.
Overall		279	253	208	298	—	284	252	229	275	—
Sex	IF	4	433	—	—	0.707	4	339	74	607	0.694
	SF	126	287	208	366		130	247	224	270	
	IM	5	260	0	606		5	266	0	595	
	CM	144	186	304			145	267	223	311	
Tumour location	Other	188	282	231	333	*0.101* ^a^	192	275	232	318	**0.009** ^b^
	Proximal humerus	91	183	110	256		92	204	160	248	
Treatment facility	Institution A (Western USA; 1997–2010)	216	263	220	306	**0.043**	217	248	218	278	0.946
	Institution B (Eastern USA; 2010–2017)	63	197	151	243		67	274	238	310	
Age	<median	159	194	143	245	*0.107*	161	161	243	225	0.877
	≥median	120	302	225	379		123	123	282	226	
Body weight	<median	147	286	235	337	0.267	149	274	216	332	0.365
	≥median	132	225	169	281		135	237	201	273	
Baseline pain	Low	82	286	246	326	0.941	84	271	220	322	0.633
	High	187	238	178	298		190	245	220	270	
Preoperative NSAID use	No	211	260	210	310	0.344	215	264	241	287	0.750
	Yes	68	237	148	326		69	235	191	279	
Preoperative non‐NSAID use	None	138	275	204	346	0.494	139	268	208	328	0.848
	Single agent	106	225	158	292		110	245	220	270	
	Multimodal	35	174	18	330		35	271	200	342	
Perioperative NSAID use	No	159	255	190	320	0.491	160	248	196	300	*0.169*
	Yes	114	260	184	336		118	252	227	277	
Intraoperative non‐NSAID use	None	0	—	—	—	0.818	0	—	—	—	0.668
	Single agent	35	249	178	320		35	243	136	350	
	Multimodal	237	270	220	320		242	255	229	281	
Postoperative non‐NSAID use	None	2	177	—	—	0.857	2	177	—	—	0.756
	Single agent	78	275	199	351		79	322	236	408	
	Multimodal	194	246	179	313		198	245	223	267	
Post‐discharge NSAID use	No	74	246	141	351	0.921	74	282	207	357	0.404
	Yes	205	255	200	310		210	250	228	272	
Post‐discharge non‐NSAID use	None	17	245	82	408	0.897	18	209	0	440	0.892
	Single agent	155	270	189	351		159	245	216	274	
	Multimodal	107	243	192	294		107	267	240	294	
NSAIDs: category of use	None	21	197	33	361	0.751	21	315	142	488	0.542
	Pre‐ and perioperative, plus post‐discharge	97	260	180	340		100	252	219	285	
Local anaesthetics: category of use	None	10	159	86	232	0.529	10	198	43	353	0.887
	Nerve or line block	141	270	216	324		143	247	212	282	
	Epidural	55	275	135	415		56	240	219	261	
	Soaker catheter	26	279	117	441		28	320	209	431	
Intensity of overall perioperative analgesic support	Low	60	243	149	337	0.290	61	252	217	287	**0.008**
	High	15	476	140	812		16	378	196	560	

Cox proportional hazard regression modelling of continuous variables	*N*	Progression‐free survival				Overall Survival			
		Risk ratio	95% Confidence Interval		Sig.	Risk Ratio	95% Confidence Interval		Sig.
			Lower	Upper			Lower	Upper	
Serum ALP activity (IU/L)	279	1.0005	1.0000	1.0009	**0.0329**	1.0002	0.9998	1.0006	0.308
Monocyte count (×10^3^/μL)	263	1.0001	0.9998	1.0005	0.3772	1.0001	0.9998	1.0004	0.556

^a^ Italicized to indicate that this variable was entered into the multivariable analysis because *p* <0.20.

^b^ Bolded for emphasis of a statistically significant result (*p* <.05).

Abbreviations:CI, confidence interval; Sig., *p* value; IF, sexually intact female; SF, spayed female; IM, sexually intact male; CM, castrated male; ALP, alkaline phosphatase, MONO, absolute monocyte count.

**TABLE 2:** Multivariable Cox proportional hazards model for prognostic and predictive factors

Comparator		N	Progression‐Free Survival		Overall survival	
			Hazard Ratio	Sig.^a^	Hazard ratio	Sig.
Treatment facility	Facility A: Western USA;	217	0.520 (0.227–1.195)	0.123	0.818 (0.417–1.605)^b^	0.560
	Facility B: Eastern USA;	67				
ALP	Continuous variable	280	1.002 (1.000–1.004)	0.073	1.001 (0.999–1.002)	0.378
Monocyte count	Continuous variable	280	1.000 (0.999–1.001)	0.832	1.000 (0.999–1.001)	0.585
Tumour location	Other	167	0.756 (0.380–1.507)	0.427	0.498 (0.287–0.866)	**0.014**
	Proximal humerus	80				
Age	Continuous variable	280	0.873 (0.787–0.969)	**0.011**	0.934 (0.863–1.012)	0.096
Baseline pain	Low	76	0.898 (0.429–1.880)	0.775	0.857 (0.480–1.530)	0.602
	High	171				
Perioperative NSAID use	No	160	1.152 (0.403–3.289)	0.792	1.346 (0.530–3.417)	0.532
	Yes	118				
Intensity of overall perioperative analgesic support	Low	61	2.748 (0.940–8.039)	0.065	2.922 (1.189–7.194)	**0.020**
	High	16				

^a^ Sig.: *p* value, Bolded for emphasis of a statistically significant result (*p* <.05).

^b^ Values inside the parentheses represent the 95% confidence interval of the hazard ratio.

To gain insight as to whether dogs getting a low intensity of overall perioperative analgesic support might have been more heavily treated with opioids, opioid usage immediately before, during, and after surgery was assessed (Table 3). All dogs received pure mu agonists during surgery, and during their inpatient care. Opioid premedications included fentanyl (4 dogs), hydromorphone (50), methadone (6), and morphine (16). Continuous rate infusions (CRI) were either fentanyl (61 dogs), morphine (1) or remifentanil (4). Whenever epidurals contained an opioid, it was morphine. Postoperatively, intermittent opioid boluses were fentanyl (16 dogs) or hydromorphone (2). Postoperatively, fentanyl CRI's were used for an average of 1.2 +/− 0.64 days in the low‐intensity group and 2.0 +/− 0.89 days in the high intensity group. Those data failed the Shapiro–Wilk normality test and were compared using the nonparametric 2‐tailed Mann–Whitney U test, wherein fentanyl CRI's were used for significantly longer (*p* <.0001) in the group having received high analgesic support. After discharge, opioids (either a transdermal fentanyl patch, oral tramadol and/or oral morphine) were prescribed to a similar proportion of dogs in each group (95% of dogs in the low analgesic support group and 93.75% of dogs in the high analgesic support group; Fisher's Exact *p* value >0.999). More dogs in the high analgesic support group were prescribed transdermal fentanyl (33% vs. 81%; *p* = .001). Subjectively, that was somewhat balanced by a higher proportion of dogs in the low‐analgesic support group having been prescribed oral morphine (22% vs. 0%; *p* = .058), meaning that pure mu opioids were prescribed in either 55% or 81% of cases, respectively (*p* = .084).

**TABLE 3:** Opioid usage in the peri‐ and postoperative periods

	Intensity of overall analgesic support		Fisher's Exact *p* value
	Low	High	
Intraoperative opioid usage			
Premedication	61/61 (100%)	15/16 (94%)	0.208
Continuous rate infusion	50/61 (82%)	16/16 (100%)	0.107
Epidural (morphine)	4/61 (7%)	6/16 (38%)	**.002** ^a^
Inpatient postoperative opioid usage			
Intermittent boluses	16/61 (26%)	2/16 (13%)	0.332
Continuous rate infusion	61/61 (100%)	16/16 (100%)	>0.999
Transdermal fentanyl	21/60 (35%)	15/16 (94%)	**<.0001**
Oral tramadol	32/60 (53%)	5/16 (31%)	0.161
Outpatient (post‐discharge) opioid usage			
Transdermal fentanyl	20/60 (33%)	13/16 (81%)	**.001**
Oral morphine	13/60 (22%)	0/16 (0%)	0.058
Oral tramadol	36/60 (60%)	9/16 (56%)	0.783

^a^ Bolded for emphasis of a statistically significant result (*p* <.05).

## 4 DISCUSSION

In this study, baseline (preamputation) osteosarcoma‐associated pain was not associated with PFS or OST. While the intensity of overall analgesic support was not associated with PFS, overall survival was prolonged in dogs receiving what was defined as a high‐intensity multimodal perioperative analgesic protocol.

A previous study reported that, in dogs treated with combinatorial chemoradiotherapy, high baseline osteosarcoma‐associated pain was associated with short overall survival.^5^ That observation was made in a relatively small sample size wherein pain severity was retrospectively classified. To build upon that observation and gain initial insight as to whether high pain may indicate a biologically aggressive tumour phenotype that is likely to metastasize sooner than a tumour with low pain, we used similar methods here to retrospectively assess this population of 284 dogs. Acknowledging the same methodologic limitations (namely that the Colorado State University acute pain scoring system has not been rigorously validated as a useful readout of pain in this disease and treatment setting, and furthermore, there has also been no validation of the methods for retrospectively bucketing baseline pain as “high” or “low”), the data and analyses presented herein do not suggest that severity of baseline tumour‐associated pain is predictive of either metastasis‐free or overall survival in dogs undergoing limb amputation plus adjunctive chemotherapy for extremity osteosarcoma. Addressing the above experimental design limitations will be required before firm conclusions can be drawn, and ideally that validation will take the form of a prospective study in which rigorous methods are used for pain assessment.

In addition to pain caused by the primary tumour, amputation is associated with pain, and later (chronic/persisting) post‐amputation neuropathic‐like pain has recently been reported to affect about one‐third of dogs.^20^ Dogs in the current study had no consistent, standardized postoperative pain assessment data available for review. Instead, we indirectly assessed postoperative pain by looking at how postoperative pain was clinically managed. This assessment of analgesic and local anaesthetic usage is limited by the fact that any measured effect may not relate to pain per se, but rather, to direct effects of analgesics. It is also limited by the fact that we only know what was given in‐hospital and what was prescribed for post‐discharge outpatient use, but we did not have any way to retrospectively determine pet owner compliance with post‐discharge instruction; data around use of NSAIDs in the home environment must be considered on an “intent to treat” basis. Finally, while we do know that the intent was to treat an included dogs with a standard course of postoperative chemotherapy, and indeed all dogs received at least one dose of carboplatin or doxorubicin, we do not know how many dogs failed to complete the prescribed course of chemotherapy. Nonetheless, this sort of analysis is capable of providing a starting point for future hypothesis‐driven research. We began by trying to understand whether use of either local anaesthetics or post‐operatively administered NSAIDs might affect progression‐free or overall survival. Local anaesthetics were of interest because they provide effective analgesia. They work by blocking voltage‐gated sodium channels on sensory neurons. Those same channels have been found in tumour cell membranes, and thus it is not surprising that lidocaine and ropivicaine have been found capable of inhibiting growth, invasion and migration of cancer cells, and directly inducing apoptosis.^21^, ^22^, ^23^, ^24^ Lidocaine may also sensitize cancer cells to cisplatin, and reverse multidrug resistance.^25^, ^26^ Additionally, local anaesthetics will prevent sensory nerve activation, and decrease the neurogenic inflammation that releases substances such as calcitonin gene‐related peptide which have the potential to influence cancer progression.^27^, ^28^, ^29^ NSAIDs were of interest because: (1) inflammation plays an important role in cancer progression;^30^ (2) long‐term NSAID use may alter the biology of some tumours;^31^ (3) perioperative NSAID administration may be associated with lower risk of local recurrence, delayed metastasis and/or prolonged survival for certain human cancers;^32^, ^33^, ^34^ and (4) for dogs, NSAIDs provide more predictable and potent postoperative analgesia as compared with other drugs that are suitable for outpatient use (e.g.,tramadol, gabapentin, and amantadine).^35^, ^36^, ^37^, ^38^ Perhaps due to small effect size, or because there simply may have been no biological difference to measure, we identified no significant associations between survival and either local anaesthetic usage, or postoperative administration of NSAIDs when assessed separately.

In line with the exploratory nature of this work, our next step was to assess other patterns of analgesic use in the perioperative period. We found no association between progression‐free or overall survival, and either: (a) preoperative use of NSAIDs or non‐NSAIDs, (b) intraoperative use of non‐NSAIDs, (b) perioperative use of NSAIDs or non‐NSAIDs, or (c) postoperative use of non‐NSAIDs. Because the overall quality of the entire multimodal perioperative analgesic plan may be more important than any individual component, our final approach involved a subset analysis in which dogs were “binned” as having received either a low or high‐intensity of perioperative analgesic support. The high‐intensity group included dogs that had received both: (1) a soaker catheter eluting local anaesthetics into the amputation site during their period of postoperative inpatient care, and (2) an NSAID during the entire perioperative period (i.e., before, during and after surgery). Dogs in the low‐intensity group did not receive an NSAID at all, and while they may have received a local anaesthetic administered as a line block, nerve block, or epidural, they did not have a soaker catheter placed at surgery and used post‐operatively. While local anaesthetics do provide excellent analgesia, their duration of action is relatively short.^39^ Longer acting liposomal formulations of bupivacaine are now available; that formulation was infrequently used in the cases reported herein and while we acknowledge the duration of analgesia may have been longer than dogs treated with tissue infiltration of lidocaine or bupivacaine, dogs treated with liposomal formulations of bupivacaine as tissue infiltrations were placed in the low analgesic group.^40^, ^41^ Soaker catheters with intermittent administration of local anaesthetics into the surgical site over a period of days following surgery are considered to provide extended periods of local analgesia.^42^, ^43^, ^44^ We found that in this population of dogs, high‐intensity analgesic support (as defined in our approach) was strongly and significantly associated with prolonged overall survival. Given that all dogs had undergone limb amputation, this suggests a difference in time to metastasis. That interpretation is limited by the fact that we evaluated all‐cause survival, rather than disease‐specific survival. We did not find a difference between the groups in terms of progression‐free (i.e., metastasis‐free) survival; however, the PFS analysis itself was limited by low quality data, which resulted from the fact that standardized restaging protocols were not in place for the cases included in this study. We did find a positive effect on overall survival from high‐intensity analgesic support and there are several possible explanations for this intriguing observation. First, it is possible that the high‐intensity multimodal analgesic support package may have reduced clinician reliance upon opioids (which have been shown experimentally to dampen anti‐tumour immunity and promote aggressive tumour behaviours such as metastasis); however, this is not supported by our data, which indicate that care for dogs in the high‐intensity group was also characterized by more frequent use of morphine‐containing epidurals, longer use of opioids delivered via continuous rate infusion postoperatively, and more frequent postoperative use of transdermal fentanyl patches.^8^, ^45^ Second, provision of effective analgesic therapy may alter (reduce) the accumulation of pro‐algesic factors (e.g., neurotrophins) in local tissues and systemic circulation; this is potentially significant because many factors which modulate pain are also known to modulate tumour behaviour.^6^, ^7^ Third, the analgesic drugs used may have had direct, combined anticancer effects on micrometastases present at the time of analgesic provision, as both NSAIDs and local anaesthetics have been shown to have anti‐cancer properties.^31^, ^46^, ^47^ Fourth, presence of pain may alter the psychological state of these animals, which can in turn alter immune function in such a way that is permissive of tumour cell growth and/or metastasis.^48^ These potential explanations are not mutually exclusive. Finally, the result could be a false positive; perhaps there is some other unmeasured or un‐appreciated factor (e.g., selection bias) that was in common for the subset of 16 dogs that had high‐intensity analgesic support.

An important potential limitation of this work is that the data were collected from two separate and geographically distant United States based veterinary hospitals. This improved sample size beyond what would have been possible with a single‐centre study, however, based on availability of case materials, one institution contributed cases from 1997 to 2010, and the other institution contributed cases from 2010–2017. All cases treated with high‐intensity multimodal analgesic support were managed at a single institution, after 2010; it is impossible to know if this is because of inherent differences in anaesthetic/analgesic practice patterns between the institutions, or because of evolving pain management practices that would have been common to both institutions. We also cannot exclude the possibility of other unmeasured confounders (e.g., it is possible that chemotherapy dose intensity varied over time or between institutions). Similarly, it is also plausible to consider that there may be hitherto unknown geographical differences in canine osteosarcoma biology.^49^

Based upon results of this study, there is no evidence that the severity of osteosarcoma‐induced bone pain at the time of presentation for treatment is a predictor or modifier of survival in dogs undergoing amputation and chemotherapy for osteosarcoma. By contrast, in a subset analysis of 77 dogs, the use of a bupivacaine‐eluting soaker catheter in combination with consistent perioperative administration of NSAIDs was associated with prolonged survival. The biologic basis for this result is unknown; regardless, the prospect of being able to prolong survival of osteosarcoma patients by optimizing perioperative analgesia is exciting. An imperative next step is external validation of these results.

## CONFLICT OF INTEREST

The authors declare no potential conflict of interest.

## Data Availability

The data that support the findings of this study are available from the corresponding author upon reasonable request.
